# Speech Intelligibility of Cochlear-Implanted and Normal-Hearing Children

**Published:** 2015-09

**Authors:** Sara Poursoroush, Ali Ghorbani, Zahra Soleymani, Mohammd Kamali, Negin Yousefi, Zahra Poursoroush

**Affiliations:** 1 *Department of Speech Therapy, School of Rehabilitation, Tehran University of Medical Sciences, Tehran, Iran.*

**Keywords:** Children, Cochlear, Implants, Language, Speech.

## Abstract

**Introduction:**

Speech intelligibility, the ability to be understood verbally by listeners, is the gold standard for assessing the effectiveness of cochlear implantation. Thus, the goal of this study was to compare the speech intelligibility between normal-hearing and cochlear-implanted children using the Persian intelligibility test.

**Materials and Methods:**

Twenty-six cochlear-implanted children aged 48–95 months, who had been exposed to 95–100 speech therapy sessions, were compared with 40 normal-hearing children aged 48–84 months. The average post-implanted time was 14.53 months. Speech intelligibility was assessed using the Persian sentence speech intelligibility test.

**Results:**

The mean score of the speech intelligibility test among cochlear-implanted children was 63.71% (standard deviation [SD], 1.06) compared with 100% intelligible among all normal-hearing children (P<0.000). No effects of age or gender on speech intelligibility were observed in these two groups at this range of ages (P>0.05).

**Conclusion:**

Speech intelligibility in the Persian language was poorer in cochlear-implanted children in comparison with normal-hearing children. The differences in speech intelligibility between cochlear-implanted and normal-hearing children can be shown through the Persian sentence speech intelligibility test.

## Introduction

Cochlear implantation is an effective treatment for hearing-impaired children whose hearing aids have not proven effective (1). However, a significant handicap for cochlear-implanted children is that they may fail to develop fully intelligible speech (2). Speech intelligibility refers to the extent of the speaker’s intended message that is understandable by listeners (3). The ultimate goal of utilizing cochlear implantation is to enable intelligible speech, because this demonstrates the communi- cation function of language (4). Thus, evaluation and treatment of speech intelligibility is an important and challenging process.

Several studies have shown that cochlear implantation is associated with improvement in speech intelligibility (5). Habib et al. (2010) found that children who were implanted in the first 24 months of life achieved higher levels of speech intelligibility. This study also showed that the age of the children affected speech intelligibility and that older children had better intelligibility scores (6). Huang et al. (2005) compared Mandarin speech intelligibility between normal-hearing and cochlear-implanted children. They showed that the differences in speech intelligibility between these two groups were statistically significant (P<0.001). They also proved that there was a positive correlation between the duration of implant usage and speech intelligibility. In addition, the age of implantation affected speech intelligibility adversely (7).

Studies on Iranian cochlear-implanted children are infrequent, especially in the field of speech intelligibility, because cochlear implant surgery has been available in Iran only for around two decades (8). Furthermore, each study needs a standardized measurement, and there was no valid or reliable intelligibility test in Persian until 2011. Thus, researchers could not study speech intelligibility in a large number of cochlear-implanted children using a standardized test.

There are a large number of English tests such as Speech Intelligibility Evaluation, The Beginner’s Intelligibility Test, and Weiss Intelligibility Test; testing at the level of the word, sentence, and conversation, respectively (9,10).

In contrast, in the Persian language, there are only two valid and reliable tests; one at the sentence level and one at the word level (11,12).

The advantages of evaluating intelligibility at the sentence level is that it is closer to the natural features of language, and better shows phonological representations and speech intelligibility in detail (13). It should be noted that certain features of sentences affect speech intelligibility in hearing-impaired children (14), including the length of a sentence; however, we found no study in which the length of sentences lead to better intelligibility scores.

The goals of our study were to evaluate the differences in speech intelligibility between normal-hearing children and cochlear-implanted, Persian-speaking children using a valid and reliable Persian test, and to evaluate the effect of sentence length on speech intelligibility.

## Materials and Methods

*Participants/ Children*

 Sixty-six children aged 48–95 months (mean age, 68.22 months; standard deviation [SD], 1.37) participated in this study. All children could speak at the sentence level and none had speech motor or behavioral problems. The children were divided into two groups. The first group included 26 cochlear-implanted children with the ages ranging from 48 to 95 months (mean, 61.30 months; SD, 1.58). The age of implantation ranged from 22 to 84 months (mean, 54.50 months; SD, 1.70). The post implantation period ranged from 9 to 26 months (mean, 14.53 months; SD, 5.27) and the subjects had been exposed to 95–100 speech therapy sessions following surgery in four hospitals in Tehran, with the same particular method at the time of testing for all of subjects. They were capable of expressing sentences. The second group included 40 normal-hearing children with the ages ranging from 48 to 94 months (mean, 67.52; SD, 1.23) as the control group. All subjects were able to express sentences. Demo graphic information is available in (Table.1). 

Four adults were taught to judge the intelligibility of the children’s speech. The average ages of these listeners was 30 years (SD,8.88). All listeners were native Persian speakers with normal hearing and were selected as unfamiliar listeners as they had no exposure to deaf or cochlear-implanted speech or any knowledge of the characteristics of speech in these children. The listeners were not allowed to listen to the sentences more than one time.

**Table 1:** Demographic characteristics of cochlear-implanted and normal-hearing children

Range of ages(months)	Number of subjects	Number of Boys	Number of Girls	Average age at implantation(months)	Post implantation period(months)
**48–59**	CI: 9 N: 13	CI: 3 N: 7	CI: 6 N: 6	CI: 36 (6.76) N:_	CI: 15.66 (5.14) N:_
**60–71**	CI: 4 N: 12	CI: 2 N: 6	CI: 2 N: 6	CI: 48.75 (9.70) N:_	CI: 15.50 (6.13) N:_
**72–83**	CI: 7 N: 9	CI: 5 N: 3	CI: 2 N: 6	CI: 64.85 (1.77) N:-	CI: 10.71 (1.70) N:_
**84–95**	CI: 6 N: 6	CI: 3 N: 3	CI: 3 N: 3	CI: 74 (8.78) N:_	CI: 16.66 (6.43) N:_

CI: Cochlear-implanted children, N: Normal-hearing children

*Speech stimuli*

 The Persian sentence speech intelligi- bility

test (see Appendix A)was utilized to assess the intelligibility of the children’s speech (11). 

**Appendix A:** Persian sentence intelligibility test lists presented phonetically

**Number**	**Sentence [In English]**	**Number of morphemes**	**Number**	**Sentence [In English]**	**Number of morphemes**
1	pesær šunemikone [The boy is combing]	5	13	tupe qermez male mæne [The red bal is mine]	7
2	doxtær midoe [The girl is running]	4	14	in tupe [This is a ball]	3
3	baba sib xord [The father ate an apple]	4	15	maman jaru kærd [Mother swept]	4
4	doxtæro maman ræftan [The mother and daughter went]	5	16	pesær zire mize [The boy is under the table]	5
5	pesær ʔænar xord [The boy ate a pomegranate]	4	17	xers ræft [The bear went]	3
6	pærænde pærvaz mikone [The bird is flying]	5	18	doxtær nešæst [The girl sat]	3
7	bæče mesvak mizæne [The child is brushing]	5	19	bæče ʔoftad [The child fell]	3
8	mahi tuye ʔabe [The fish is in the water]	5	20	in šire [This is a lion]	3
9	tup nist [The ball is not here]	3	21	mahi šena mikone [The fish isswimming]	5
10	tup ʔoftad [The ball felt]	3	22	Maman xabid [Mother slept]	3
11	In mašin kučike[This car is small]	4	23	ʔab rixt [Water spilt]	3
12	maman jaru mikone [The mother is sweeping]	5			

This test was designed for use in Persian-speaking children aged 4 to 7 years with hearing loss. The test involved 23 sentences and 67 words. Each sentence was simple, and could be represented through pictures and videos. Sentences in the test contained words familiar to children. Each sentence included between two and seven words and between three and seven morphemes. Test-retest reliability was 0.99 and Cronbach’s alpha coefficient was 0.98. The content validity index of all 23 sentences was 1. The differential validity of the test was −0.70. The concurrent validity was 0.83. This testis divided into four groups according to the number of morphemes (Table. 2). 

**Table2:** Characteristics of Persian sentence intelligibility test according to number of morphemes

Groups	Number of morphemes	Number of sentences
**1**	3	9
**2**	4	5
**3**	5	8
**4**	7	1

*Test administration*

All 23 sentences in this test were administered to the children. During the test administration, a speech therapist conveyed the target sentence to the child in any way, for example using objects or videos or saying the sentence, and the participant repeated the sentences. The entire assessment session for each child was audio recorded by a digital recorder in a quiet room, and all children were tested separately. The recordings were then digitized to preserve all 23 sentences spoken by the child. All sentences were saved in a computer in preparation for the listening session (Table. 2).

*Listening session*

The listening session was conducted by the four listeners described above. The listeners could choose the intensity of the voice recording. They listened to the sentences in a quiet room and wrote down what they had heard.

In order to prevent a learning effect, the sentences were presented randomly and the listeners received no feedback on their judgment. Listeners wrote down what they had heard immediately after playing back each sentence.

*Ethics*

The study was approved by the Medical Ethics Committee of Tehran University of Medical Sciences. The aims and procedures of the study were explained to all parents, and signed informed consent was provided.

*Data analysis*

 Data were analyzed using the Statistical Package for Social Sciences (SPSS), version 16.0. The written responses were scored based on the percentage of correctly transcribed words. All 67 words were scored,and the words were weighted equally.

The first analysis examined differences in speech intelligibility between the cochlear-implanted children and normal-hearing children. A t-test was applied to compare the speech intelligibility scores of these groups. 

In the second analysis, the effect of sentence length on speech intelligibility was examined.

The sentences were sorted based on the number of morphemes. A one-way analysis of variance (ANOVA) was applied to compare speech intelligibility of the four sentence groups. All statistical analyses were performed using a significance level of P<0.05.

## Results

The results of the sentence intelligibility test showed that for the 26 cochlear-implanted children, speech intelligibility scores ranged from 47.69-100% (mean, 63.71; SD, 1.06). The corresponding score for all 40 normal-hearing children was 100% (Table. 3).

**Table 3:** Descriptive statistics of cochlear-implanted children and normal-hearing children

Range of ages (months)	Average age (SD)	Rang of intelligibility	Average intelligibility scores (SD)
**48–59**	CI: 51.66 (3.87) N: 54.15 (3.69)	CI: 56–100	68.52 (1.47) 100 (0.00)
**60–71**	CI: 64.25 (4.19) N: 65.75 (3.27)	CI: 58.46–63.07	60.76 (1.97) 100 (0.00)
**72–83**	CI: 76.57 (2.87) N: 75.55 (3.67)	CI: 55.38–66.15	60.87 (3.85) 100 (0.00)
**84–95**	CI: 90.66 (3.77) N: 88.00 (3.79)	CI: 47.69–76.92	61.79 (1.14) 100 (0.00)

CI: Cochlear-implanted children, N: Normal-hearing children

The difference in speech intelligibility between normal-hearing and cochlear-implanted children was statistically significant (P<0.000), as shown in (Table 4). There was no significant difference in speech intelligibility in terms of age in the cochlear-implanted group (F_(3,22)_=0.94; P=0.43) or the normal group. Only one of the cochlear-implanted children reached 100% speech intelligibility.

There was a significant difference between groups in speech intelligibility in terms of different morpheme sentences (F_(3,99)_=4.26; P=0.007). The sentences with three and seven morphemes only showed a significant difference (P<0.004) in the *post hoc* test.

**Table 4:** Differences in Intelligibility

**Group**	**Mean**	**Standard deviation**	**t**	**df**	**Sig(2-tailed)**
Cochlear-implanted	63.71	1.06	-21.43	63	0.000
Normal-hearing	100	0	-17.43	25	

## Discussion

In this study, speech intelligibility was measured in 26 cochlear-implanted children who had been exposed to 95–100 speech therapy sessions in the previous 9 to 26 months and then compared with that of 40 normal-hearing children. The study showed a significant difference in speech intelligibility between cochlear-implanted and normal-hearing children; as expected, speech intelligibility scores were higher for the normal-hearing group. There are several possible explanations for this finding, for example cochlear-implanted children do not correctly articulate 60–70% of consonants in the first year of using the cochlear implant (15), and do not have the same auditory experience as normal children (16). We would expect greater improvement in speech intelligibility when the child has used the cochlear implant for a longer period of time (17). 

This study supports previous studies conducted in this area. For example, Huang compared the speech intelligibility of cochlear-implanted children with normal-hearing children at the level of words, consonants, vowels, and tones and found the differences to be statistically significant (7). 

Flipsen compared the intelligibility of conversational speech in six children under the age of 3 years. He reported that conversational speech in cochlear-implanted children is better than that in children using hearing aids, but not necessarily as good as in children with normal hearing (2).

This study showed no correlation between age and speech intelligibility. Because access to auditory information via cochlear implants is an important factor for sound repertoires and speech intelligibility and all of these children had the same period of hearing and auditory feedback (18,19). Speech intelligibility reached 100% in the normal-hearing children at 48 months (20,21) and all of normal-hearing children in this study were 48–95 months years old. The effects of age shown in this study are consistent with other studies; Huang et al. reported that speech intelligibility was not correlated with age in cochlear-implanted children (7).

Our study showed no difference in speech intelligibility between girls and boys in the two groups. This finding is consistent with previous studies. Branan and Ghasisn 2010 found no difference between speech intelligibility in normal-hearing girls and boys (21,22). Since the sequence of phonetic acquisition and phonetic classes is the same in the two groups, this may reflect the similarity in the intelligibility of boys and girls (21). 

Only one of the cochlear-implanted children scored 100% in the intelligibility test. This child was 49 months years old and received the implant at 29 months (a younger age than all other participants except one who was implanted at 22 months). In addition, this child had undergone 100 speech therapy sessions in 20 months. There are numerous factors which affect speech intelligibility, such as higher nonverbal intelligence, socialization, training in learning environment, and extensive parents support (23-25). This child had probably been exposed to these extra factors.

This study showed a significant difference between seven- and three-morpheme sentences; the intelligibility of sentences that have seven morphemes was higher than those of three morphemes. One reason for this observation is that longer sentences have more linguistic-contextual features and it is easier for the listeners to utilize linguistic-contextual knowledge of sentences in order to understand the utterances (26). However, we cannot be sure that this observation is due specifically to the length of sentence because intelligibility is also related to word position, length and fluency of utterance, phonological complexity, syllabic structures and the grammatical form of sentence (9). It may be these that factors contributed to this finding.

## Conclusion

The results of this study lead us to the conclusion that it is difficult for cochlear-implanted children to reach full speech intelligibility after 1 or 2 years using a cochlear implant. However, it is not impossible. This study mandates similar studies with a longer period time after implantation, in order to show the effect of the cochlear implant in the development of speech intelligibility. In the future studies, evaluation of other factors such as word position, length and fluency of utterance, phonological complexity, syllabic structures and the grammatical form of sentences should be considered.
